# Giant cell tumor of axial vertebra: surgical experience of five cases and a review of the literature

**DOI:** 10.1186/s12957-015-0438-4

**Published:** 2015-02-18

**Authors:** Guojing Chen, Jing Li, Xiangdong Li, Hongbin Fan, Zheng Guo, Zhen Wang

**Affiliations:** Department of Orthopedics, Xijing Hospital, Fourth Military Medical University, Changle Road, Xi’an, 710032 People’s Republic of China

**Keywords:** Giant cell tumor, Axial vertebra, Intralesional, Reconstruction, Odontoid process

## Abstract

**Background:**

Due to the complex anatomy of the upper cervical spinal column region and the variable aggressiveness of giant cell tumors (GCTs), there exists no standard treatment for GCTs of axial vertebra. To the best of our knowledge, there are only a few case reports in the literature and no large sum numbers of clinical trials about the treatment of, or research into, axial vertebra GCTs.

**Methods:**

Between 2009 and 2013, five patients pathologically diagnosed with axial vertebra GCTs were treated at our hospital. We performed intralesional excision and odontoid process reconstructive surgery to preserve the odontoid process, followed with adjuvant radiation therapy after surgery.

**Results:**

For those with an intact bone shell, part of the β-TCP (beta tricalcium phosphate) artificial bone could be seen clearly after surgery and became blurred three months after surgery, as seen on a radiograph. One year later, the part of β-TCP artificial bone was fused as a block. Subsequently, autogenous bone regenerated successfully and artificial bone degraded thoroughly. For those with a defective cortical bone, partial fusion of the odontoid process, autograft ilium and third vertebra body could be seen three months after surgery, and complete fusion was seen nine months later. The odontoid process was preserved successfully, and the upper cervical spine was reconstructed effectively, without implant failure or infection.

**Conclusions:**

In this study, the odontoid process and function of upper cervical vertebra was preserved successfully through lesion curettage, combined with reconstruction with bone grafting, and adjuvant radiation therapy after surgery. During the follow-up periods, no recurrence or complications was observed.

## Background

Giant cell tumors of the bone (GCTs), are one of the most common benign primary bone tumors, and are well known for their locally aggressive behavior and tendency to recur [1]. With a slight predominance in females compared to males, GCTs accounted for approximately 5% of bone tumors in adults between 20 and 50-years-old globally [2,3]. After the knee and the radius, the sacrum is the third most common location of GCTs in bones [4,5] and the most common location in the axial skeleton. Primary GCTs of the cervical spine account for only about 2 to 3% of spine tumors, and GCTs in the axial vertebra (C2) are especially rare [6,7].

Although generally considered a benign tumor, 5 to 10% of GCTs are reported as malignant and aggressive tumors [8,9]. The aggressiveness of the tumor led to local recurrence in 7 to 75% of patients [9,10]. Of reported GCTs, 1 to 9% metastasize to the lungs despite the fact that the primary lesion was histologically considered to be benign [11]. If the tumor is located in a complex and important region, such as upper cervical spinal column, local recurrence and distal aggressiveness of the tumor often has a serious causality and a high risk of fatality.

Due to the complex anatomy of the upper cervical spinal column region and the variable aggressiveness of GCTs, there is no standard treatment procedure for GCTs of the spinal column, especially in atlas (C1) and axial vertebra (C2). Traditionally, treatment options for GCTs included wide resection (Ec bloc), intralesional curettage with or without adjunctive techniques [12,13] and radiation therapy [14]. Radiation eliminates surgical complications to a great extent but may cause local injuries, such as early and late skin injuries, neuritis, secondary pathologic fractures and so on [13,14]. Wide resection was theoretically intended to prevent local recurrence, but it increases surgical morbidity with complications such as hematoma, functional deficits, skin sloughs and fistulas, deep infection and problems resulting from reconstruction for the anatomic structure and biomechanics stability [15]. Local curettage of the lesion preserves the integrity of the column, however, the local recurrence rate is especially high, ranging from 6.3 to 43% [12,13]. The scarcity of reports in the available literature on this topic provides limited protocol for clinicians to achieve maximum local control and minimum surgical complications in patients with axial vertebra GCTs. However, whether to preserve the odontoid process of the axial vertebra or not is substantially important to the anatomy structure and function preservation of the atlantoaxial vertical joint.

There are only a few case reports in the literature and no large sum numbers of clinical trials about the treatment of, and research into, axial vertebra (C2) GCTs. However, to the best of our knowledge, there have been only a few reports of the preservation of the odontoid process for axial vertebra tumors [16,17]. Between 2009 and 2013, five patients pathologically diagnosed with axial vertebra GCTs were treated at our hospital. We performed intralesional excision and odontoid process reconstructive surgery to preserve the odontoid process, followed with adjuvant chemotherapy after surgery. We introduce the surgical experience of five cases and evaluate retrospectively the effectiveness of this treatment for axial vertebra GCTs in preserving anatomy structure and function of the atlantoaxial vertical joint.

## Methods

### Basic characteristics

We retrospectively reviewed the records of all five patients who had a primary GCT involving the axial vertebra and who were treated with surgery between 2009 and 2013. There were two male and three female patients, with a mean age of 31 years (23 to 45 years) (Table 1). Three patients were treated with intralesional surgery and two patients underwent partial resection to preserve the odontoid process, all followed with adjuvant radiation therapy after surgery. The mean follow-up period was 31.4 months (10 to 48 months) for all patients. All five GCTs were located in a single segment of the axial vertebra.We had prior ethics approval from Institutional ethical committee of Xijing Hospital (NO: 20090135) and patient consent was obtained for this study. WBB (Weinstein-Boriani-Biagini anatomic zone classification for tumor) stages of tumor and Frankel grades of nerve functions are shown in Table 2.

**Table 1:** **Basic characteristics of the patients**

**Case**	**Age**	**Gender**	**Location of the tumor**	**Surgery**	**Follow-up**
1	27	Male	C2	intralesional curettage	48 months
2	36	Female	C2	intralesional curettage	37 months
3	24	Female	C2	intralesional curettage	10 months
4	45	Female	C2	partial resection	34 months
5	23	Male	C2	partial resection	28 months

C2: axial vertebra.

**Table 2:** **Clinical information and results of all five patients**

**Case**	**WBB stages**	**Frankel grade**		**Pain (VAS)**		**Recurrence**
		**Pre-op**	**3 months** ^**a**^	**Pre-op**	**3 months** ^**a**^	
1	4-9/B-D	E	E	6	2	NO
2	4-9/B-D	E	E	7	3	NO
3	4-9/B-D	E	E	5	2	NO
4	4-9/A-D	D	E	6	1	NO
5	4-9/B-D	D	E	4	1	NO

^a^Post-operation. NO: no recurrence during the follow up periods; Pre-op: pre-operation; VAS: Visual Analogue Scale.

The research was carried out according to the principles set out in the Declaration of Helsinki 1964 and all subsequent revisions. Informed consent was obtained and the relevant institutional review board had approved the study.

### Clinical information and imaging

**Figure 1:**
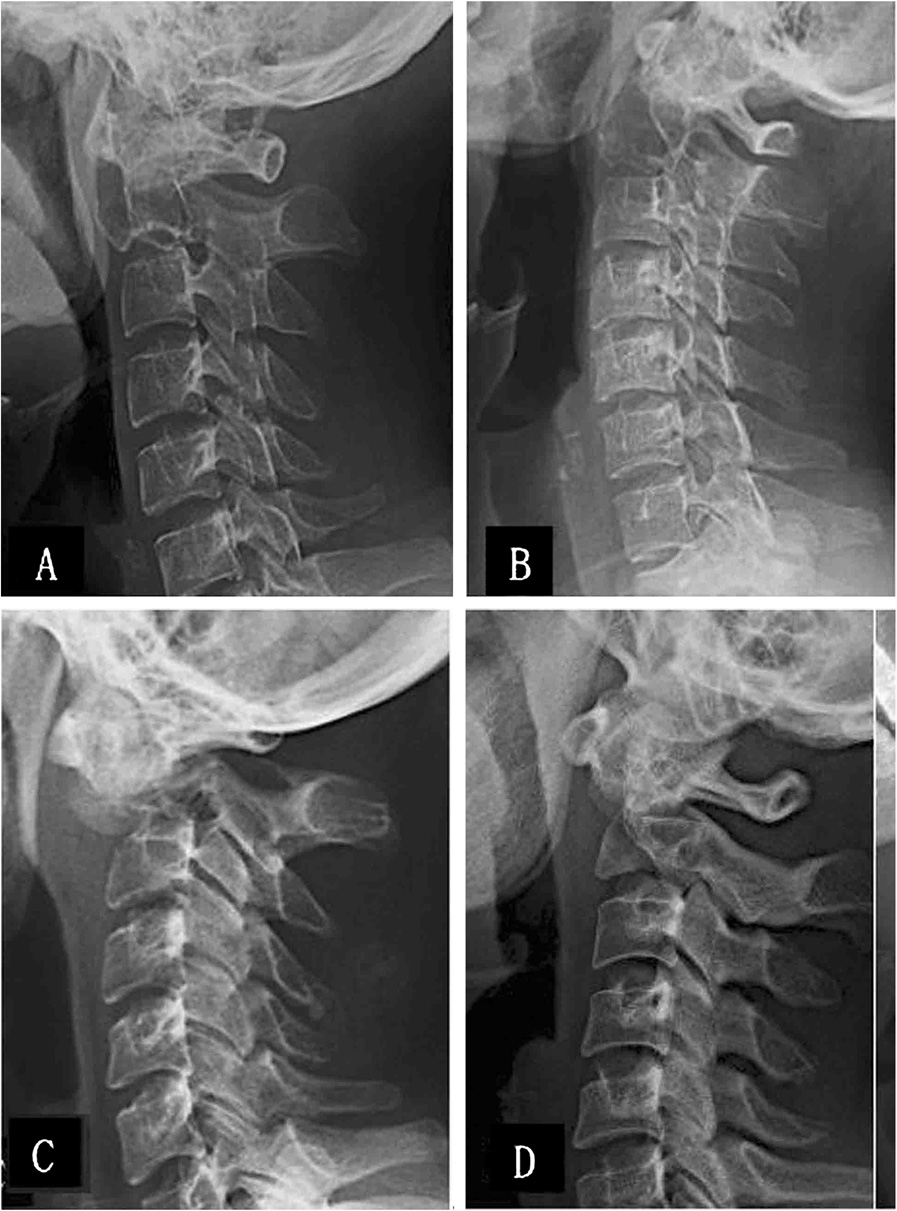
**Preoperative view: radiograph characteristics of the pathological axial vertebra (C2).** Expansile osteolysis of the axial vertebra with giant cell tumors (GCTs) and intact cortical bone of the axial vertebra affected **(A, B)**. Pathological fractures of the odontoid process and sub-dislocation of the atlantoaxial vertical joint **(C, D)**. GCTs invaded the cortical bone of the axial vertebra (C2) and a soft tissue mass was seen around **(C)**.

**Figure 2:**
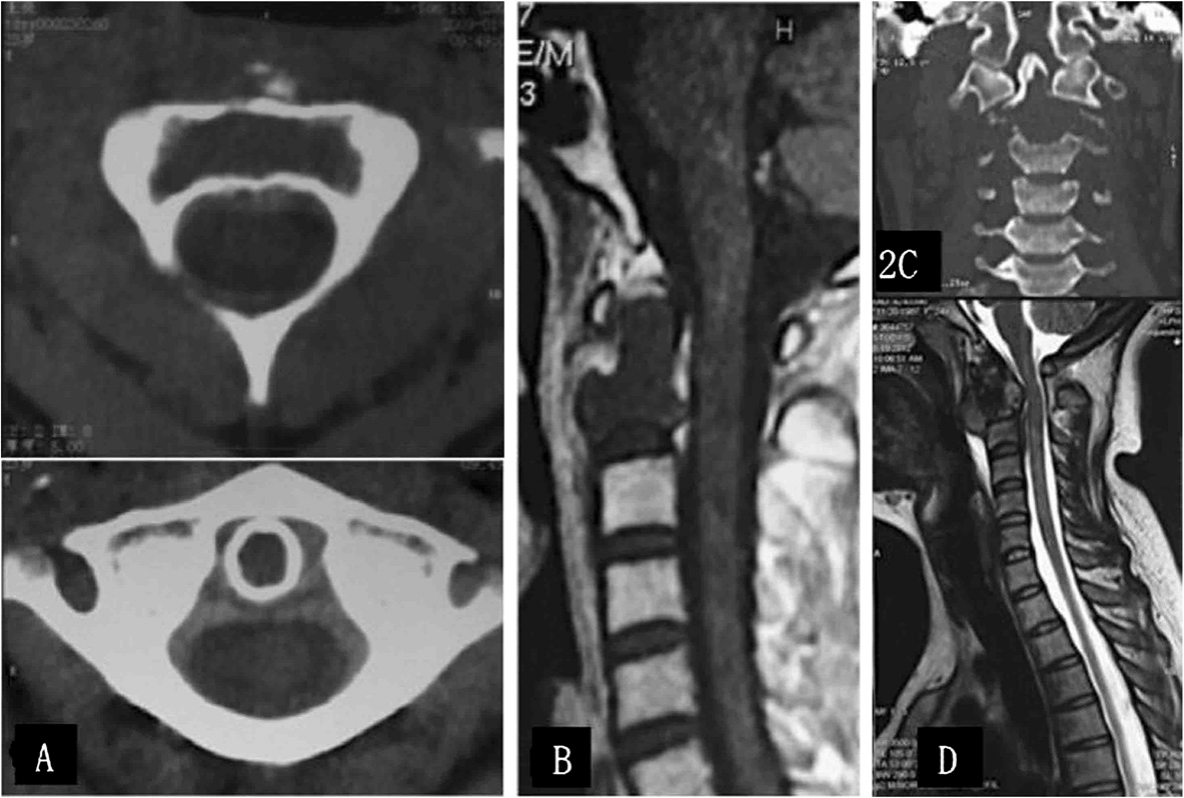
**Computed tomography scans showing osteolysis destruction of the axial vertebra (C2) with giant cell tumors (GCTs) and the intact cortical bone of the axial vertebra odontoid process. (A, C)** Magnetic resonance imaging showing the GCTs that invaded the cortical bone of the axial vertebra and the soft tissue mass that was seen around **(B, D)**. Tumors and the soft tissue mass are showed as low signal imaging on T1-WI.

The diagnosis of axial vertebra GCT was established based on clinical information and imaging studies (Figures 1 and 2), and confirmed by pathology examination after surgery. All five patients with GCTs experienced different scales of pain locally in the upper cervical spine and the pain became increasingly serious. Two patients felt numbness or pain intensively in the upper limb and had to take non-steroidal anti-inflammatory drugs orally to relieve the pain (case two and case four). Two other patients took on the symptoms or signs of cervical vertebrae instability and spinal cord compression, such as shaky gait, hypertonia of the lower extremity muscular tension, hyperreflexia of patellar tendon, Babinski’s sign (+) and Hoffmann's sign (+) (case one and case three). One of the five patients experienced limited motion of the cervical vertebrae suddenly after a trivial trauma. Subsequently, she suffered from both dyspnea and dysphagia (case five).

The stage of the GCTs were set radiographically according to the system of Campanacci [18] as follows: an intraosseous lesion (Grade I); an intraosseous lesion with cortical thinning and expansile borders (Grade II) and an extraosseous lesion, forming a soft tissue mass (Grade III). There were two patients with Grade I lesions, two patients with Grade II lesions and one with Grade III lesions.

### Surgical methods

**Figure 3:**
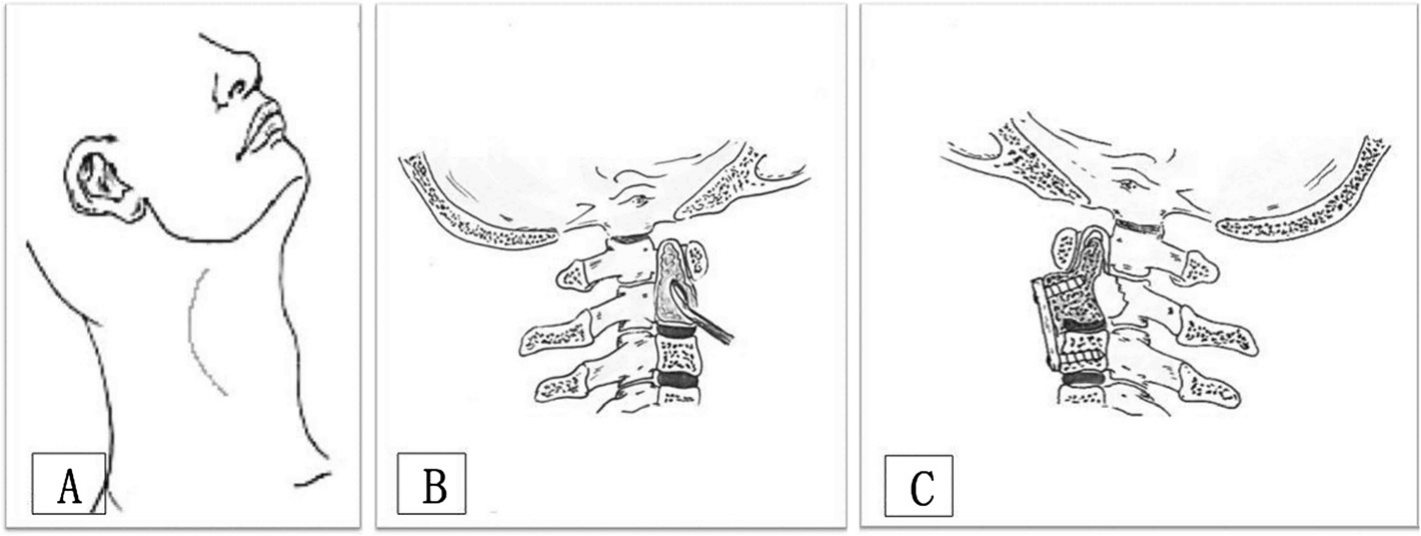
**Surgical approach and skin incision.** Skin incision **(A)**. Intralesional curettage for those with an intact bone shell of the odontoid process and preservation of the odontoid process **(B)**. Partial resection of the axial vertebral body and autograft ilium for those with a defective cortical bone of the axial vertebra **(C)**.

**Figure 4:**
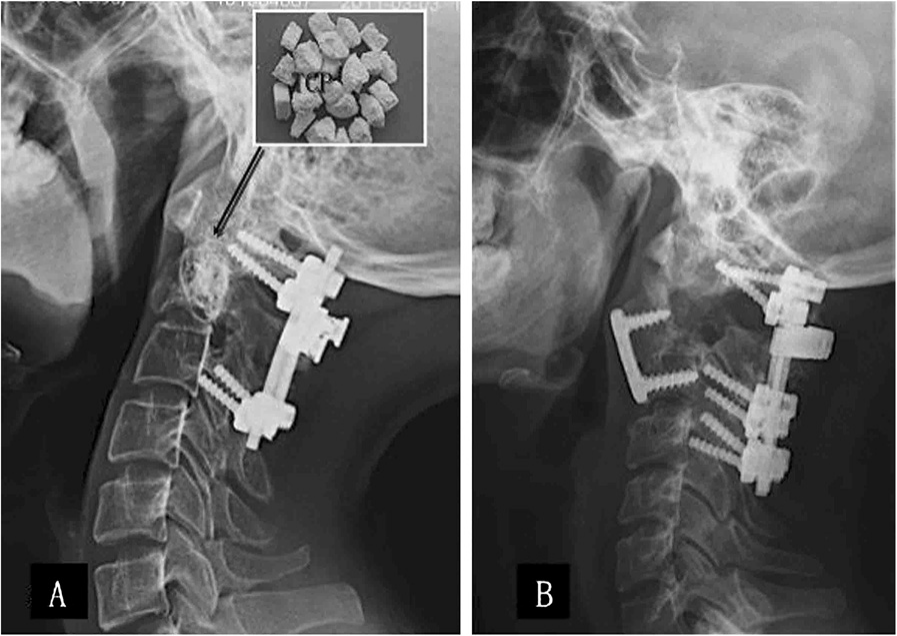
**Follow-up radiograph.** For those with an intact bone shell, part of the β-TCP artificial bone was implanted in the cavity for reconstruction of the axial vertebra. Forty-eight months post-operation, autogenous bone had regenerated successfully and artificial bone had degraded thoroughly **(A)**. For those with a defective cortical bone of the axial vertebra, an autograft ilium was implanted and made up for the defect of the axial vertebra body that had been resected. Twelve months post-operation, the odontoid process and autograft ilium were shown to have fused completely on a radiograph **(B)**. No recurrence was observed in both cases during the follow-up periods.

All surgeries were performed by one surgeon under general endotracheal anesthesia. Arterial blood pressure was monitored and a deep venous cannula was intubated continuously during the periods of operations. For all five patients, we fixed the cervical spine through the posterior approach, combined with lesion curettage through the anterior approach. Astride across the axial vertebra (C2), the pedicle of the atlas vertebral arch (C1) and the lateral mass of the third vertebra (C3) were fixed together stably using cervical pedicle screw systems (Medtronic, Minneapolis, USA) through the posterior approach in three cases (cases one, two and three). For the anterior approach, the axial vertebra (C2) was exposed through inner margin approach of the sternocleidomastoid muscle at the inferior angle of the mandible (Figure 3A). For those with an intact bone shell, surgical treatment options were thorough intralesional curettage and preservation of the odontoid process (Figure 3B). Then the base of lesion was cauterized using 50% zinc chloride for 20 minutes and irrigated three times with large volumes of saline. The β-TCP artificial bone (Bio-Lu Bioceramics , Shanghai, China) was implanted (Figure 4A) in the cavity for reconstruction of the axial vertebra (C2) [19].

To make sure the fixation was firm, the pedicle of the atlas vertebral arch (C1) and the lateral mass of third/fourth vertebra (C3 and C4) were fixed together using cervical pedicle screws through the posterior approach in the two cases with pathological fractures of the odontoid process (case four and case five). For those with a defective cortical bone of the axial vertebra (C2), the axial vertebral body was resected and the odontoid process was preserved with thorough intralesional curettage through the anterior approach (Figure 3C). Then, the autograft ilium was cut and trimmed in a ‘kitchen knife’ outline form, so that the ‘knife holder’ inserted into the odontoid process preserved and the ‘knife body’ made up for the defect of the axial vertebra body that had been resected. For the fusion of the odontoid process, autograft ilium and third cervical vertebra body, a cervical spinal plate (Medtronic, Minneapolis, USA) was implanted through the anterior approach (Figure 4B). To improve local control of the GCT after surgery, three-dimensional conformal radiation therapy was administered (total dose: 30 to 456 Gy) as an adjuvant treatment one week after incision healing for all five patients.

## Results

The mean operation duration was 120 minutes (range: 90 to 180 minutes). The total blood loss was 400 ml (range: 200 to 800 ml) and most of the blood was lost during the course of intralesional curettage. The mean follow-up period was 31.4 months after surgery. Nerve root pain and local pain eased substantially without any analgesic one month after surgery. Pain disappeared almost three months after surgery. The Visual Analogue Scale (VAS:0-10 scores) scores for pain were shown in Table 2. Muscle strength and muscle tension returned to normal and neural function as measured by the Frankel grade decreased from D to E at three months after surgery (Table 2). No recurrence was observed in all five cases during the follow-up periods. The patient was symptom free in all five cases at the last time of follow-up.

For those with an intact bone shell, part of the β-TCP artificial bone could be seen clearly after surgery and became blurred three months after surgery as seen on a radiograph. One year later, a radiograph showed that the part of the β-TCP artificial bone had fused as a block. Subsequently, autogenous bone had regenerated successfully and artificial bone had degraded thoroughly (Figure 4A). For those with a defective cortical bone of the axial vertebra (C2), partial fusion of the odontoid process, autograft ilium and third vertebra body could be seen three months after surgery, and complete fusion could be seen nine months later on a radiograph (Figure 4B). The odontoid process was preserved successfully and the upper cervical spine was reconstructed effectively without implant failure or infection in all five cases. Stability and activity of the cervical spine was satisfactory for all patients.

## Discussion

GCTs of the bone were classified by the World Health Organization (WHO) as a benign but locally aggressive tumor [20]. They occur most frequently in young adults between 20 and 40 years of age [21-23]. A higher incidence of GCTs has been reported for the Chinese population, in which it can account for up to 20% of all bone tumors [21,24].

Currently, there is no consensus regarding the ideal treatment selection for all patients with GCTs. Various studies suggest that wide resection provides the lowest recurrence rate of GCTs [21,25]. However, wide resection is associated with higher rates of surgical complications [26,27] and is often accompanied by considerable functional impairment [28]. Instead, intralesional excision is a preferable treatment when feasible with the aim of excising the whole tumor and sparing the native joint [28-30]. However, curettage alone has been associated with a high rate of local recurrence [26,31]. Therefore, various adjuvants have been employed to ‘extend’ the curettage [30,32] and no clear evidence exists as to whether adjuvant therapies are most effective [33,34]. The fact that there is no single treatment to control the recurrence of the lesion effectively has created a lot of studies on GCTs. In this clinical study, we describe our experience on treating GCTs of the axial vertebra (C2) and discuss the effectiveness and safety of upper cervical spine reconstruction following intralesional curettage surgery.

Recently, it has been widely reported that tumors of the spine are managed successfully with *en bloc* resections whenever possible, as this provides the greatest chance for cure [35,36]. However, *en bloc* resections are almost impossible for the axial vertebra (C2) because of the complicated anatomy and the important biomechanical functions of it. The anatomy of the upper cervical spinal column region contains the vertebral artery, cervical nerve root, cervical spinal cord and atlantoaxial vertical joint. According to Enneking’s classification for bone tumors [37], GCTs of the vertebral column are classified as Enneking stage three of the spine benign tumor, that is to say, an aggressive benign tumor. Theoretically, intralesional excision is one of the feasible treatments for axial vertebra GCTs. In this clinical study, intralesional curettage, combined with bone grafting, and adjuvant radiation therapy after surgery, was an effective therapeutic strategy for all five cases with GCTs. However, only skillful surgeons would be able to practice those complicated surgical techniques with skill and ease as it requires experience to master the surgical approach, lesion resection and function reconstruction of the upper cervical vertebral column.

Due to the anatomic characteristics of the upper cervical vertebral column, the anterior approach is the most common surgical technique for the lesion resection of axial vertebra (C2) in the literature [16,17]. It has also been reported that trans-oral approach surgery or splitting the mandible approach surgery are frequently used techniques to expose the axial vertebra (C2). The pathological axial vertebra could be exposed directly and easily through these approaches, however, there are numerous avoidable disadvantages. Firstly, it is technically difficult to control severe bleeding due to the limited operating space in the trans-oral approach. Secondly, it increases the disunion incidence of mucous membrane, because the posterior wall of the pharynx membrane was is thin to contain the grafting bone and/or internal implants. Thirdly, it increases the contamination rate of incision due to the non-aseptic atmosphere of the oral cavity. Lastly, reconstruction of mandible is technically difficult and would present dysfunction of chewing following the splitting the mandible approach. However, the approach in our study overcame the disadvantages of the trans-oral approach and the splitting the mandible approach. Through the inner margin of the sternocleidomastoid muscle and the inferior angle of the mandible incision, the axial vertebra (C2) was exposed distinctly and the lesion was curetted successfully. There were also advantages such as the small size of incision, the low incidence rate of complication and so on. Due to the complicated structure of this approach, the adjacent superior laryngeal nerve, hypoglossal nerve and submandibular gland should be protected cautiously.

For all five patients, we fixed the cervical spine through the posterior approach and then curetted the lesion through the anterior approach. In three of the patients who had an intact cortex, the odontoid process was preserved successfully using 50% zinc chloride and radiation therapy. This verifies the hypothesis that 50% zinc chloride is effective in decreasing the recurrence of GCTs. In two of the patients who had a defective cortical bone, the odontoid process was preserved with an axial vertebral body resection and the axial vertebra was reconstructed with an ilium autograft. For the fusion of the odontoid process, autograft ilium and third cervical vertebra, it was necessary to implant a cervical spinal plate through the anterior approach. Consistent with a previous study [19], either β-TCP artificial bone or autograft ilium bone, depending on the defection volume of the pathological bone, were the potential biological implants.

To stabilize the reconstruction after axial vertebral body resection, it has been reported that atlanto-occipital joint fixation and posterior fusion is the most frequently used surgical technique in the literature. Without a doubt, the function of the atlanto-occipital joint is almost lost after fixation and fusion. However, it is not necessary to fix the atlanto-occipital joint, especially in cases where the atlas vertebra and odontoid process are intact. In our study, the pedicle of the atlas vertebral arch (C1) and lateral mass of the third/fourth vertebra (C3 and C4) were fixed together instead. This not only immobilized the C1-C3 efficiently, but also greatly preserved the structure of odontoid process and function of atlanto-occipital joint. Also, we completed the treatment of GCTs of the axial vertebra by the posterior and anterior approaches in one stage, which was a time- and cost-effective way for the patients.

Due to the complex anatomy of the axial vertebra and the variable aggressiveness of GCTs, it was substantially difficult to resect the tumor mass and to eliminate scattered tumor cells. Thus, a suitable adjuvant therapy seemed necessary to control the tumor cell scatter. In studies by Chakravarti *et al*. [38] and Shi W *et al*. [39], it was demonstrated that adjuvant radiation therapy could control the recurrence of GCTs, however, it also induced serious radiation myelopathy. Nevertheless, the use of three-dimensional conformal radiation therapy altered the adverse reaction of radiation therapy. Further, accurate localization and adjustable dose prevented the spinal cord from radiation injury. In our study, no recurrence or complication was observed during the follow-up periods.

## Conclusions

There are only a few case reports in the literature and no large numbers of clinical trials about treatment of, and research into, axial vertebra (C2) GCTs. In this study, the odontoid process and function of upper cervical vertebra was preserved successfully through lesional curettage, combined with reconstruction with bone grafting, and adjuvant radiation therapy after surgery. During the follow-up periods, no recurrence or complications related were observed. Due to the small sample size of this study, the results should be examined cautiously. Larger, high-quality clinical trials are required to strengthen and verify these conclusions.

## Abbreviations

GCTs: giant cell tumors
β-TCP: beta tricalcium phosphate
C1: The 1^st^ Cevical vertebra
C2: The 2^nd^ Cevical vertebra
C3: The 3^rd^ Cevical vertebra
VAS: Visual Analogue Scale
